# On ten species of jumping spiders from Xishuangbanna, China (Araneae, Salticidae)

**DOI:** 10.3897/zookeys.1062.72531

**Published:** 2021-10-14

**Authors:** Cheng Wang, Shuqiang Li

**Affiliations:** 1 Guizhou Provincial Key Laboratory for Biodiversity Conservation and Utilization in the Fanjing Mountain Region, Tongren University, Tongren, Guizhou 554300, China Tongren University Tongren China; 2 Institute of Zoology, Chinese Academy of Sciences, Beijing 100101, China Institute of Zoology, Chinese Academy of Sciences Beijing China

**Keywords:** East Asia, morphology, new species, supplement, taxonomy

## Abstract

Nine new species of jumping spiders from Xishuangbanna Tropical Botanical Garden are described: *Euochinyaoi***sp. nov.** (♀♂), *Laufeiabanna***sp. nov.** (♀♂), *Marengotangi***sp. nov.** (♀♂), *Myrmarachneliui***sp. nov.** (♀♂), *Nandiciusproszynskii***sp. nov.** (♂), *Phintelloidespengi***sp. nov.** (♀♂), *Poecilorchesteszhengi***sp. nov.** (♀♂), *Rhenewandae***sp. nov.** (♂) and *Simaethacheni***sp. nov.** (♀♂). The female of *Chinattusinflatus* Wang & Li, 2020 is described for the first time.

## Introduction

China is second only to Brazil in jumping spider species diversity, with 560 species in 121 genera (Li 2020; Metzner 2021; WSC 2021). The taxonomy of these species, however, is plagued by more than 40% (223 species) of species known only from a single-sex, with many species lacking diagnostic drawings (WSC 2021).

Surveys of spiders in Xishuangbanna Tropical Botanical Garden (XTBG) have revealed 782 spider species (Li 2020). The total number of XTBG jumping spider species, including the new ones here, is 137 (Cao et al 2016; Lin and Li 2020; Wang and Li 2020a, b) with about 45% endemicity, exceeding the number of salticid species known from the entirety of Vietnam and is nearly equal to the number of species found in Japan (Metzner 2021).

The goal of the present paper is to describe new taxa based on recent specimens collected from XTBG. The female of *Chinattusinflatus* Wang & Li, 2020 is also described.

## Materials and methods

Specimens were collected by fogging and sieving leaf litter in the tropical rainforest of Xishuangbanna Tropical Botanical Garden, Yunnan, China, which were preserved in 75% ethanol for morphological study. All specimens are deposited in the Institute of Zoology, Chinese Academy of Sciences (IZCAS) in Beijing, China. Methods follow those of Wang and Li (2020a, b).

All measurements are given in millimeters. Leg measurements are given as: total length (femur, patella + tibia, metatarsus, tarsus). References to figures in the cited papers are listed in lowercase type (fig. or figs); figures in this paper are noted with an initial capital (Fig. or Figs).

Abbreviations used in the text and figures are as follows:

**AERW** anterior eyes row width;

**AME** anterior median eye;

**ALE** anterior lateral eye;

**AG** accessory gland;

**AR** atrial ridge of epigyne;

**AS** anterior chamber of spermatheca;

**At** atrium of epigyne;

**BP** basal plate of epigyne;

**CD** copulatory duct;

**CO** copulatory opening;

**CP** cymbial process;

**DTA** dorsal tibial apophysis;

**E** embolus;

**EC** embolic coil;

**EFL** eye field length;

**FD** fertilization duct;

**H** epigynal hood;

**LP** lateral epigynal plate;

**MS** median septum;

**PED** process of embolic disc;

**PERW** posterior eye row width;

**PL** posterior lobe of bulb;

**PLE** posterior lateral eye;

**PS** posterior chamber of spermatheca;

**RFA** retrolateral femoral apophysis;

**RTA** retrolateral tibial apophysis;

**S** spermatheca;

**SD** sperm duct;

**TB** tegular bump;

**TE** tooth of embolic base;

**TF** tibial flange.

Institutional abbreviations:

**IZCAS**Institute of Zoology, Chinese Academy of Sciences;

**XTBG**Xishuangbanna Tropical Botanical Garden, Chinese Academy of Sciences.

## Taxonomy

### Family Salticidae Blackwall, 1841

#### Genus Chinattus

Taxon classificationAnimaliaAraneaeSalticidae

Logunov, 1999

06DBDBB6-D56B-58E1-8F71-FDC9E3E2F3B8

##### Type species.

*Habrocestoidesszechwanensis* Prószyński, 1992 from China.

#### Chinattus inflatus

Taxon classificationAnimaliaAraneaeSalticidae

Wang & Li, 2020

656DAEBC-B9AF-5116-B0C1-5665F9550F19

Fig. 1

Chinattus inflatus Wang & Li, 2020b: 49, figs 3A–D, 4A–D (D♂)

##### Type material examined.

***Holotype*** ♂ (IZCAS-Ar40604), China: Yunnan: Xishuangbanna, Mengla County, Menglun Town, XTBG, tropical rainforest (21°55.20'N, 101°16.21'E, ca. 550 m), 30.iv.2019, Y. Tong leg. ***Paratype*** 1♂ (IZCAS-Ar40605), same data as holotype.

##### Other material examined.

1♀ (IZCAS-Ar42566), same data as holotype; 1♀ (IZCAS-Ar42567), same locality, 07.viii.2018, C. Wang leg.

##### Diagnosis.

**Figure 1.**
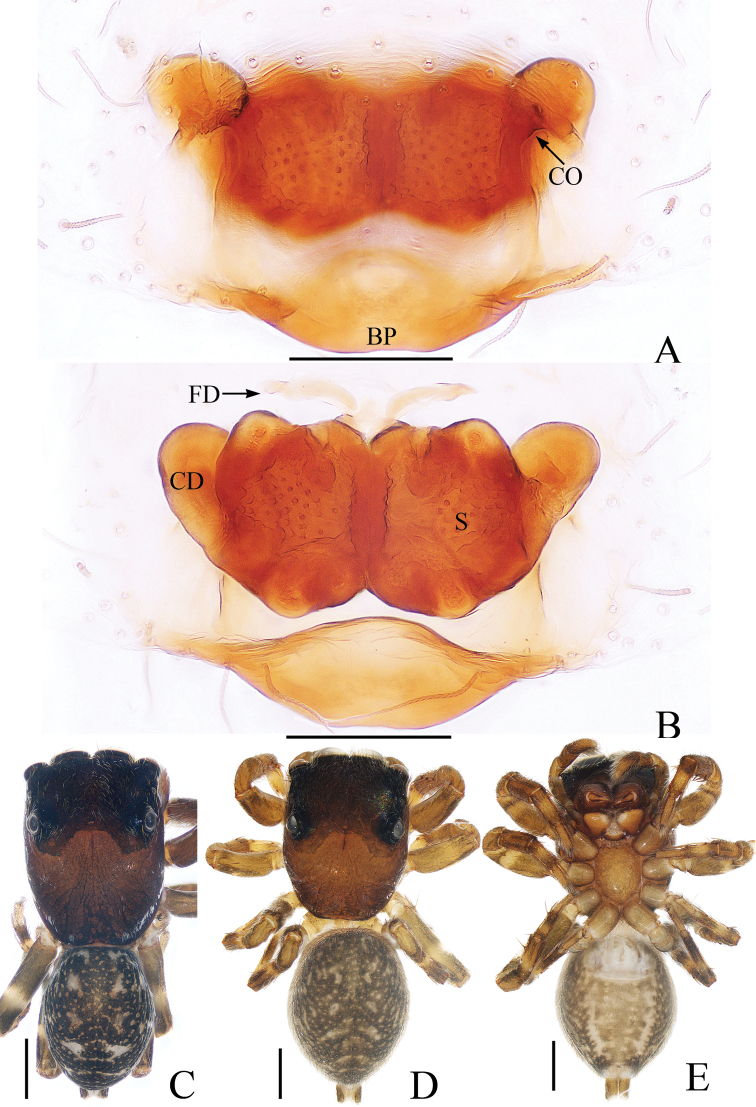
Male holotype and female of *Chinattusinflatus***A** epigyne, ventral **B** vulva, dorsal **C** holotype habitus, dorsal **D** female habitus, dorsal **E** ventral. Scale bars: 0.1 (**A, B**); 0.5 (**C–E**).

The male was thoroughly diagnosed in Wang and Li (2020b). The female of this species closely resembles that of *C.chichila* Logunov, 2003 in having a similar epigyne, but it differs in the following: 1) the basal epigynal plate has a bow-shaped posterior margin (Fig. 1A) vs. V-shaped margin in *C.chichila* (Logunov 2003: figs 3, 4); 2) the copulatory ducts are connected to the spermathecae laterally (Fig. 1B) vs. connected dorsally to the posterior edges of the spermathecae in *C.chichila* (Logunov 2003: figs 5, 6).

##### Description.

**Male**. Complete description in Wang and Li (2020b).

**Female** (Fig. 1A, B, D, E). Total length 3.24. Carapace 1.59 long, 1.23 wide. Abdomen 1.54 long, 1.21 wide. Clypeus 0.09 high. Eye sizes and inter-distances: AME 0.37, ALE 0.24, PLE 0.21, AERW 1.20, PERW 1.18, EFL 0.74. Legs: I 2.76 (0.85, 1.10, 0.48, 0.33), II 2.42 (0.83, 0.93, 0.33, 0.33), III 3.06 (1.03, 1.05, 0.65, 0.33), IV 3.19 (1.03, 1.05, 0.78, 0.33). Carapace yellow-brown to dark brown, covered with brown and white, thin setae. Fovea red-brown, bar-shaped, longitudinal. Chelicerae red-yellow. Endites broadened meso-distally, pale ental margins. Labium darker than endites, paler distally. Sternum nearly shield shaped, yellow-brown, with thin, brown setae. Legs yellow to yellow-brown. Abdomen oval, dorsum gray-brown, with irregular pale dots and transverse stripes posteriorly, covered with short, thin setae; venter brown, laterally with a pair of pale longitudinal stripes extending from epigastric furrow to terminus.

Epigyne (Fig. 1A, B): wider than long; basal plate almost equal in width to epigyne, with a bow-shaped posterior margin; copulatory openings C-shaped, located laterally; copulatory ducts curved almost 180° medially, connected to spermathecae laterally; spermathecae almost square, touching medially; fertilization ducts anterior to spermathecae.

##### Distribution.

China (Yunnan).

##### Comments.

The pairing of this species with the female is supported by DNA barcoding (unpubl. data).

#### Genus Euochin

Taxon classificationAnimaliaAraneaeSalticidae

Prószyński, 2018

2993B7BA-3014-5B53-B17D-9FF040219821

##### Type species.

*Euophrysatrata* Song & Chai, 1992 from China.

#### Euochin yaoi sp. nov.

Taxon classificationAnimaliaAraneaeSalticidae

4DD59B88-7643-5362-9AB1-E4A7ABF87564

http://zoobank.org/79AD82B9-C615-4DA0-97B3-766E537A6FF7

Figs 2 , 3

##### Type material.

***Holotype*** ♂ (IZCAS-Ar42568), China: Yunnan: Xishuangbanna, Mengla County, Menglun Town, secondary tropical seasonal moist forest (21°54.72'N, 101°16.94'E, ca. 660 m), 5–12.ix.2006, G. Zheng leg. ***Paratypes*** same locality and collector of the holotype, 1♀ (IZCAS-Ar42569), 5–12.x.2006; 1♀ (IZCAS-Ar42570), 19–25.x.2006; 1♀ (IZCAS-Ar42571), 5–12.xii.2006; 1♀ (IZCAS-Ar42572), 19–25.xii.2006; 1♀ (IZCAS-Ar42573), 19–25.i.2007; 1♀ (IZCAS-Ar42574), 19–25.ii.2007; 1♀ (IZCAS-Ar42575), 1–15.iii.2007; 1♀ (IZCAS-Ar42576), 1–15.v.2007; 1♀ (IZCAS-Ar42577), 10–20.06.2007; 1♂ (IZCAS-Ar42578), Bamboo plantation off G213 roadside (21°53.65'N, 101°16.98'E, ca. 590 m), 26.xi.2009, G. Tang, Z. Yao leg.; 1♀ (IZCAS-Ar42579), secondary tropical montane evergreen broad-leaved forest (21°54.77'N, 101°11.43'E, ca. 880 m), 1–15.i.2007, G. Zheng leg.; 1♂ (IZCAS-Ar42580), primary tropical seasonal rainforest (21°57.67'N, 101°11.89'E, ca. 640 m), 4–11.v.2007, G. Zheng leg; 1♀ (IZCAS-Ar42581), primary tropical seasonal rainforest (21°57.67'N, 101°11.89'E, ca. 640 m), 1–15.vii.2007, G. Zheng leg.

##### Etymology.

The specific name is a patronym in honor of Prof. Zhiyuan Yao (Shenyang, China), one of the collectors of the new species; noun (name) in genitive case.

##### Diagnosis.

**Figure 2.**
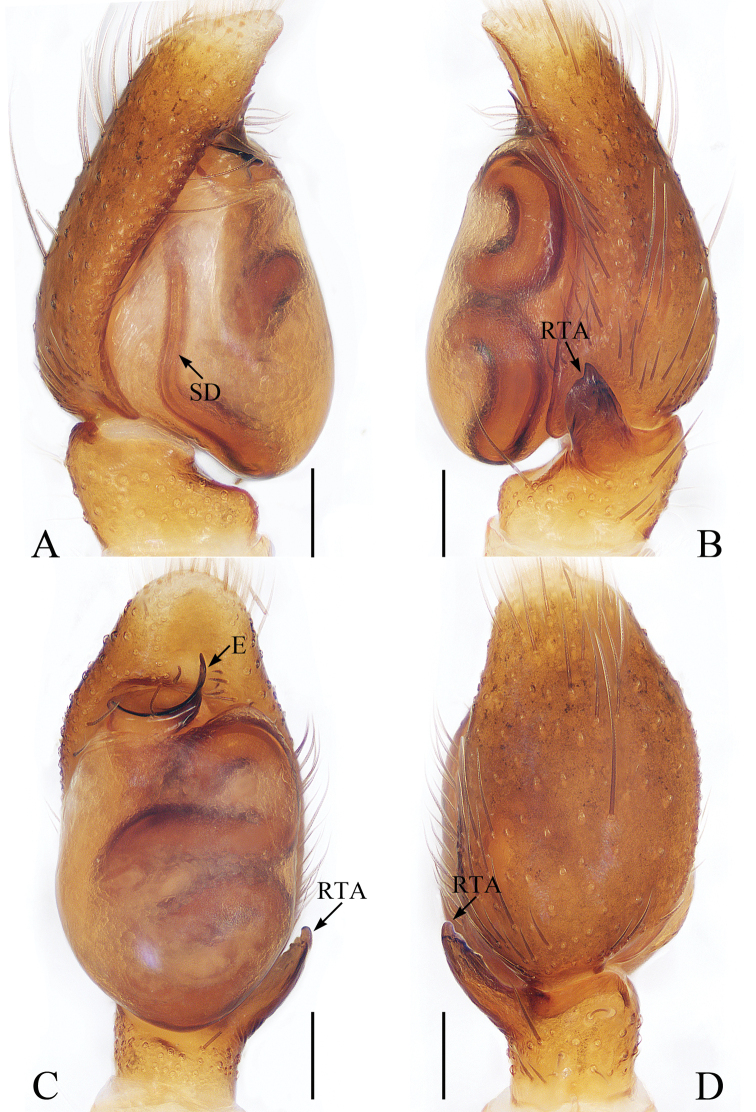
Male palp of *Euochinyaoi* sp. nov., holotype **A** prolateral **B** retrolateral **C** ventral **D** dorsal. Scale bars: 0.1.

**Figure 3.**
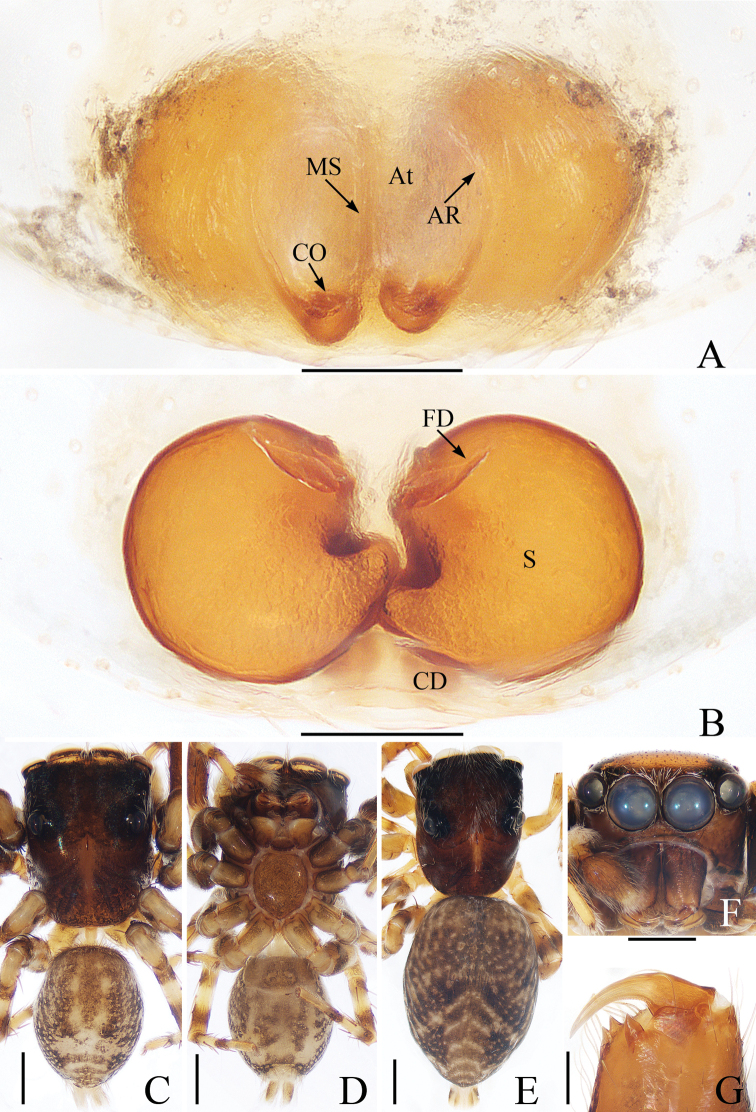
Female paratype and male holotype of *Euochinyaoi* sp. nov. **A** epigyne, ventral **B** vulva, dorsal **C** holotype habitus, dorsal **D** ventral **E** female paratype habitus, dorsal **F** holotype carapace, frontal **G** holotype left chelicera, posterior. Scale bars: 0.1 (**A, B, G**); 0.5 (**C–F**).

The male of *Euochinyaoi* sp. nov. resembles that of *E.subwanyan* (Wang & Li, 2020) in having a flat embolus and oval bulb, but it can be easily distinguished from the latter by the retrolateral tibial apophysis, which is about 1.5× longer than wide and acutely narrowed to a pointed tip distally in retrolateral view, and by the shorter embolus (Fig. 2B, C). In contrast, the RTA is almost 3× longer than wide and tapered to a blunted tip in retrolateral view, and the embolus is longer in *E.subwanyan* (Wang and Li 2020a: fig. 5B, C). The female is similar to *Euophryslongyangensis* Lei & Peng, 2012 by the posteriorly located copulatory openings and sub-spherical spermathecae, but it can be easily distinguished from the latter by having a distinct atrium and by the curved copulatory ducts, which are longer than the fertilization ducts (Fig. 3A, B) vs. indistinct and with straight copulatory ducts that are markedly shorter than the fertilization ducts in *E.longyangensis* (Lei and Peng 2012: fig. 5d, e).

##### Description.

**Male** (Figs 2, 3C, D, F, G). Total length 3.24. Carapace 1.68 long, 1.24 wide. Abdomen 1.39 long, 1.08 wide. Clypeus 0.06 high. Eye sizes and inter-distances: AME 0.39, ALE 0.26, PLE 0.25, AERW 1.13, PERW 1.08, EFL 0.77. Legs: I 3.22 (1.01, 1.23, 0.60, 0.38), II 2.85 (0.91, 1.05, 0.51, 0.38), III 3.26 (1.10, 1.10, 0.68, 0.38), IV 3.59 (1.13, 1.18, 0.88, 0.40). Carapace red-brown to dark brown, with a narrow, longitudinal, yellow-red stripe extending across nearly entire thorax centrally, covered with white and brown setae. Fovea red-brown, bar-shaped, longitudinal. Chelicerae yellow to red-brown, with 1 retromarginal tooth and 2 promarginal teeth. Endites yellow to dark yellow, distal end pale entally, with brown setae. Labium darker than endites, linguiform. Sternum almost shield-shaped. Legs yellow to red-brown, legs III, IV with annuli. Abdomen oval, dorsum dotted laterally, with pair of fitful longitudinal yellow stripes anteromedially, irregular transverse bands posteriorly; venter colored as dorsum.

Palp (Fig. 2A–D): femur about 2 times longer than wide; tibia wider than long, with a ventral protuberance and a flat retrolateral apophysis acutely narrowed to a pointed tip distally in retrolateral view; cymbium about 1.7 times longer than wide, and gradually narrowed at distal 1/3 in ventral view; bulb inflated, almost oval; embolus flat, originating from the anterior portion of bulb, coiled nearly 360°, with a pointed tip directed anteriorly in ventral view.

**Female** (Fig. 3A, B, E). Total length 3.67. Carapace 1.55 long, 1.16 wide. Abdomen 2.13 long, 1.50 wide. Clypeus 0.07 high. Eye sizes and inter-distances: AME 0.40, ALE 0.26, PLE 0.23, AERW 1.24, PERW 1.13, EFL 0.82. Legs: I 2.91 (0.93, 1.15, 0.48, 0.35), II 2.76 (0.90, 1.03, 0.48, 0.35), III 3.32 (1.08, 1.13, 0.73, 0.38), IV 3.60 (1.13, 1.20, 0.87, 0.40). Habitus similar to that of male except dorsum of abdomen lacks yellow stripes.

Epigyne (Fig. 3A, B): wider than long; atrium oval, with a pair of arc-shaped lateral ridges, separated by a narrow median septum; copulatory openings at posterior portion of atrium; copulatory ducts extending anteriorly, connecting to postero-ental portion of spermathecae; spermathecae almost spherical, separated by less than 1/6 their diameter; fertilization ducts originating from the antero-ental portion of spermathecae, lamellar.

##### Distribution.

Known only from the type locality in Yunnan, China.

##### Comments.

The species is placed in this genus based on the flat embolus, the dense, long, white setae on the proximal half of the cymbium and distally on the male palpal tibia (mostly missing in the holotype), and the copulatory ducts are markedly shorter than the spermathecal diameter; these characters are unique to *Euochin* species (Prószyński et al. 2018; Logunov 2020). However, the spherical spermathecae, posterior origin of the copulatory ducts, and the single retromarginal cheliceral tooth differ from those of the type species of *Euochin* (oval spermathecae, copulatory ducts originate anteromedially, and with retromarginal cheliceral fissident), indicating that further data are required to confirm the placement of this species. The male and female are considered to be conspecific because they were collected from the same localities and share similar habitus markings.

#### Genus Laufeia

Taxon classificationAnimaliaAraneaeSalticidae

Simon, 1889

DF7C656B-D5DD-52D3-822D-DD11B4B227BE

##### Type species.

*Laufeiaaenea* Simon, 1889 from Japan.

#### Laufeia banna sp. nov.

Taxon classificationAnimaliaAraneaeSalticidae

C1AADF86-8ACA-5B4D-9B56-347A30A60344

http://zoobank.org/02601950-9E58-4EF1-B924-19CBAC308F8C

Figs 4 , 5

##### Type material.

***Holotype*** ♂ (IZCAS-Ar42582), China: Yunnan: Xishuangbanna, Mengla County, Menglun Town, XTBG, Lvshilin (21°54.61'N, 101°16.89'E, ca. 640 m), 14.xi.2009, G. Tang leg. ***Paratype*** 1♀ (IZCAS-Ar42583), same data as holotype.

##### Etymology.

The species name is derived from the name of the type locality; noun in apposition.

##### Diagnosis.

**Figure 4.**
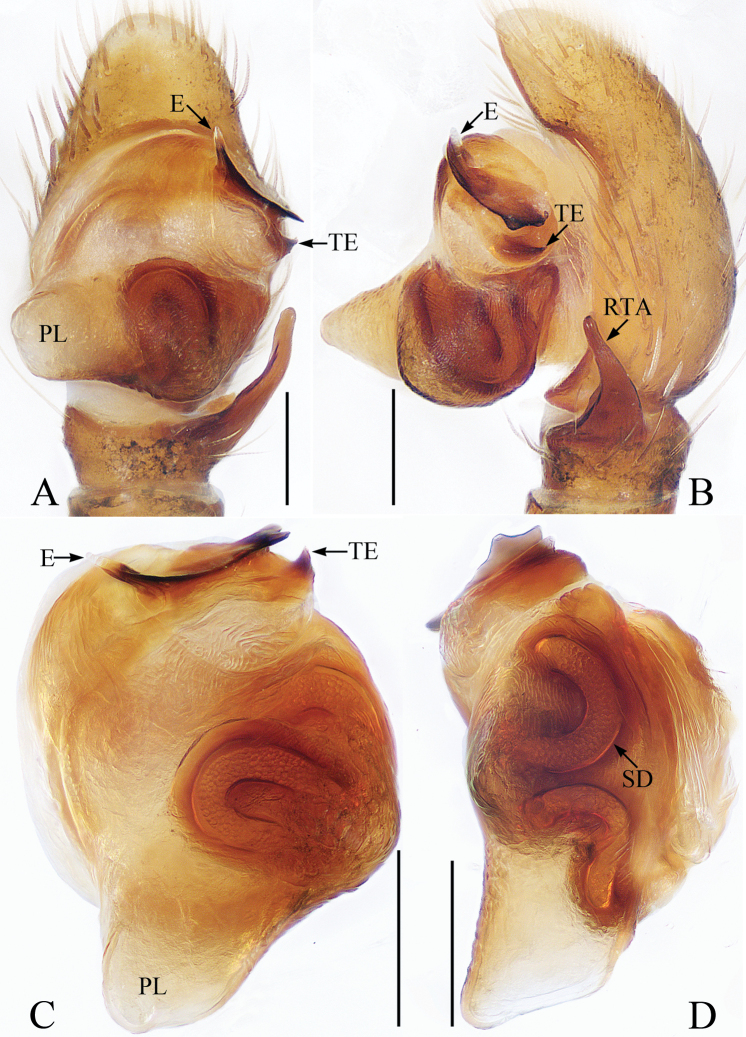
Male palp of *Laufeiabanna* sp. nov., holotype **A** ventral **B** retrolateral **C** bulb, ventral **D** retrolateral. Scale bars: 0.1.

**Figure 5.**
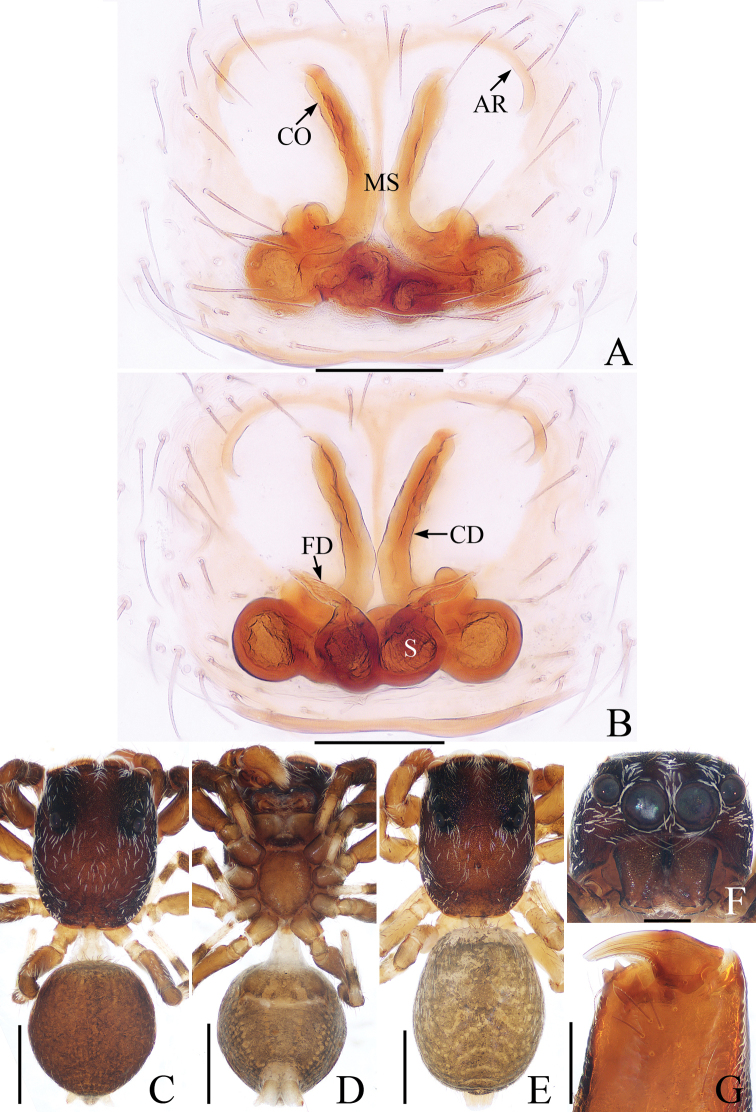
Female paratype and male holotype of *Laufeiabanna* sp. nov. **A** epigyne, ventral **B** vulva, dorsal **C** holotype habitus, dorsal **D** ventral **E** female paratype habitus, dorsal **F** holotype carapace, frontal **G** holotype left chelicera, posterior. Scale bars: 0.1 (**A, B, G**); 0.5 (**C–E**); 0.2 (**F**).

The species closely resembles that of *L.aenea* Simon, 1889 in having a branched embolic projection and medially inflated copulatory ducts, but it differs in having: 1) the embolus longer than the tooth of the embolic base in ventral view (Fig. 4A, C) vs. much shorter than the tooth of the embolic base in *L.aenea* (Ikeda 1998: fig. 6); 2) the retrolateral tibial apophysis is slightly curved (Fig. 4A, B) vs. twisted in *L.aenea* (Ikeda 1998: figs 5, 6); 3) the atrium has a pair of arc-shaped, anterolateral ridges (Fig. 5A) vs. only one anterior ridge in *L.aenea* (Ikeda 1998: fig. 7); 4) the copulatory openings are elongated and slit-shaped (Fig. 5A) vs. oval in *L.aenea* (Ikeda 1998: fig. 7).

##### Description.

**Male** (Figs 4, 5C, D, F, G). Total length 2.11. Carapace 1.04 long, 0.79 wide. Abdomen 0.88 long, 0.83 wide. Clypeus 0.01 high. Eye sizes and inter-distances: AME 0.22, ALE 0.14, PLE 0.13, AERW 0. 68, PERW 0.63, EFL 0.42. Legs: I 1.50 (0.53, 0.55, 0.21, 0.21), II 1.36 (0.45, 0.48, 0.21, 0.22), III 1.46 (0.50, 0.50, 0.23, 0.23), IV 1.59 (0.50, 0.58, 0.28, 0.23). Carapace red-brown, darker laterally and at bases of PLEs and PMEs, covered with sparse, thin, white setae, slightly denser on face. Fovea almost round. Chelicerae red to dark brown, with 2 promarginal teeth and 1 retromarginal fissident with 2 cusps. Endites broadened mediodistally, pale ental margins. Labium linguiform, paler distally. Sternum somewhat shield shaped. Legs brown to dark brown, except tarsi yellow, covered with sparse, white setae. Abdomen almost spherical, dorsum red-brown, with several transverse dotted lines posteriorly, covered by sparse, pale, thin setae; venter brown, with 4 longitudinal dotted lines.

Palp (Fig. 4A–D): femur about 2 times longer than wide; tibia wider than long, with tapered retrolateral apophysis, broadened at base, slightly curved medially and blunt apically; cymbium longer than wide, with brown setae and several white scales; bulb sub-oval, with well-developed posterior lobe; embolus spinose, membranous distal half, with pointed, branched projection, and short, tapered basal tooth.

**Female** (Fig. 5A, B, E). Total length 2.27. Carapace 1.06 long, 0.80 wide. Abdomen 1.14 long, 0.90 wide. Clypeus 0.02 high. Eye sizes and inter-distances: AME 0.23, ALE 0.15, PLE 0.13, AERW 0. 70, PERW 0.64, EFL 0.45. Legs: I 1.52 (0.48, 0.58, 0.23, 0.23), II 1.41 (0.45, 0.50, 0.23, 0.23), III 1.54 (0.53, 0.53, 0.25, 0.23), IV 1.71 (0.55, 0.63, 0.30, 0.23). Habitus similar to that of male except pale and with a longer abdomen.

Epigyne (Fig. 5A, B): almost as long as wide, with pair of arc-shaped anterolateral ridges; copulatory openings elongated, slit-shaped, anteromedially located; copulatory ducts extending posteriorly, then inflated into a sphere and continue extending transversely to connect to dorsum of spermathecae; spermathecae sub-spherical, touching; fertilization ducts anterior to spermathecae, directed anterolaterally.

##### Distribution.

Known only from the type locality in Yunnan, China.

#### Genus Marengo

Taxon classificationAnimaliaAraneaeSalticidae

Peckham & Peckham, 1892

7C0C33A9-0979-599E-A5C2-2578DFA604A7

##### Type species.

*Marengocrassipes* Peckham & Peckham, 1892 from India.

#### Marengo tangi sp. nov.

Taxon classificationAnimaliaAraneaeSalticidae

46BA7622-3B7B-5E41-B38B-160C32F593B0

http://zoobank.org/75419E1C-FD88-460F-87BC-16D5DE7A3C3F

Figs 6 , 7

##### Type materials.

***Holotype*** ♂ (IZCAS-Ar42584), China: Yunnan: Xishuangbanna, Mengla County, Menglun Town, XTBG, Tropical evergreen rainforest (21°56.21'N, 101°16.20'E, ca. 560 m), 1.xii.2009, G. Tang leg. ***Paratypes*** 12♂ (IZCAS-Ar42585–42596), same date as holotype; 2♀ (IZCAS-Ar42597–42598), site #1 of garbage dump off G213 roadside (21°54.28'N, 101°16.75'E, ca. 630 m), 25.iv.2019, Z. Bai leg.

##### Etymology.

The species name is a patronym in honor of Dr. Guo Tang, one of the collectors of the new species; noun (name) in genitive case.

##### Diagnosis.

**Figure 6.**
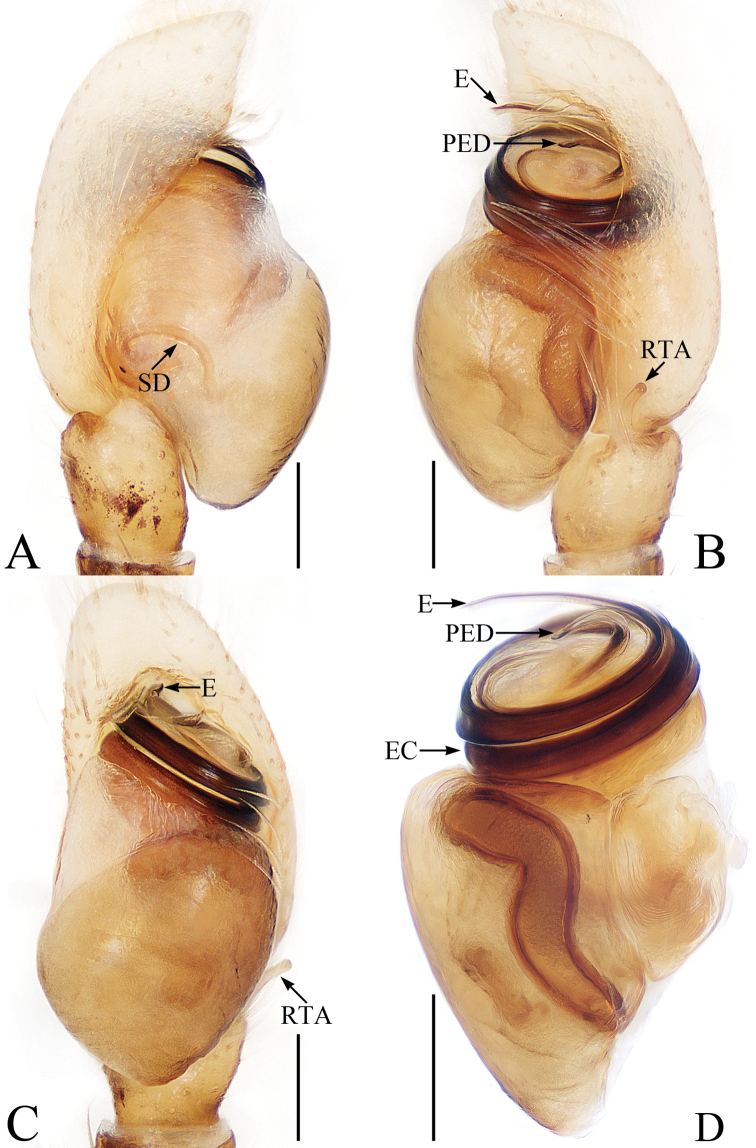
male palp of *Marengotangi* sp. nov., holotype **A** prolateral **B** retrolateral **C** ventral **D** bulb, retrolateral. Scale bars: 0.1.

**Figure 7.**
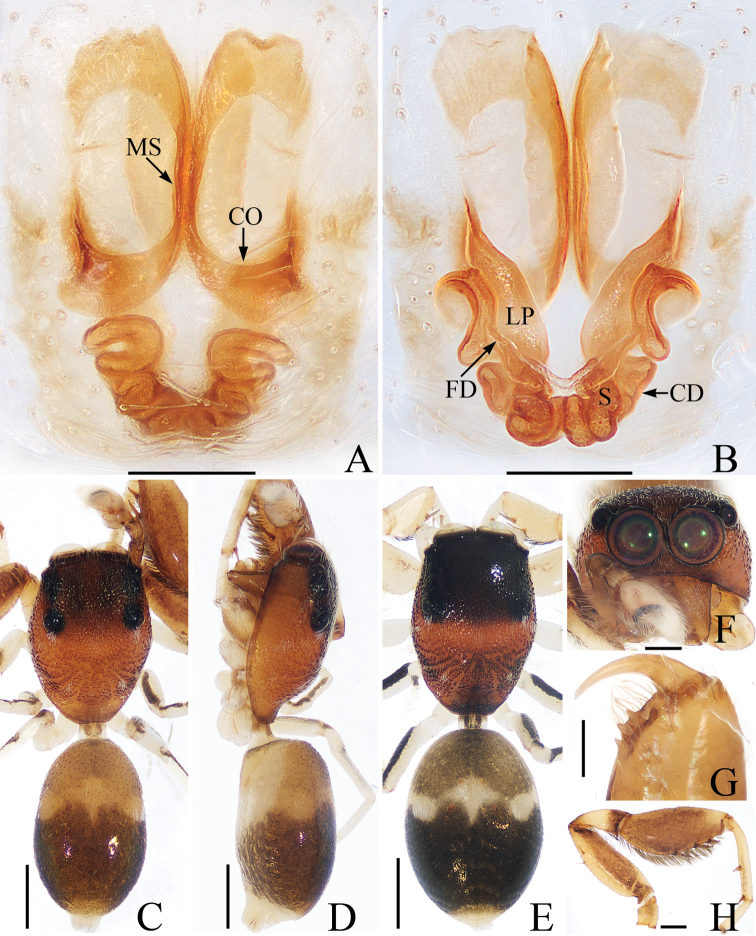
Female paratype and male holotype of *Marengotangi* sp. nov. **A** epigyne, ventral **B** vulva, dorsal **C** holotype habitus, dorsal **D** lateral **E** female paratype habitus, dorsal **F** holotype carapace, frontal **G** holotype left chelicera, posterior **H** holotype leg I, prolateral. Scale bars: 0.1 (**A, B, G**); 0. 5 (**C–E, H**); 0.2 (**F**).

The male of *Marengotangi* sp. nov. closely resembles that of *M.batheryensis* Sudhin, Nafin, Benjamin & Sudhikumar, 2019 by the similar abdominal markings and retrolateral tibial apophysis but differs by the following: 1) the process of the embolic disc is conspicuous in retrolateral view (Fig. 6B, D) vs. indistinct in *M.batheryensis* (Sudhin et al. 2019: figs 29, 41); 2) the tibiae I are about 2.3× longer than wide (Fig. 7H) vs. about 1.6× in *M.batheryensis* (Sudhin et al. 2019: figs 37, 38); 3) the scutum only covers the dorsal half of the abdomen in lateral view (Fig. 7D) vs. nearly covers the entire abdomen in *M.batheryensis* (Sudhin et al. 2019: fig. 35). The female is similar to *M.striatipes* Simon, 1900 by having a similarly-shaped habitus and elongate-oval atrium, but it can be distinguished by having a pair of lateral epigynal plates, and by the atrium about 2 times longer than wide (Fig. 7A, B), vs. absent, about 1.5 times longer than wide in *M.striatipes* (Benjamin, 2004: 67B, C).

##### Description.

**Male** (Figs 6, 7C, D, F–H). Total length 2.82. Carapace 1.27 long, 0.99 wide. Abdomen 1.43 long, 0.89 wide. Clypeus 0.07 high. Eye sizes and inter-distances: AME 0.32, ALE 0.13, PLE 0.11, AERW 0.79, PERW 0.80, EFL 0.55. Legs: I 3.44 (1.08, 1.10, 0.88, 0.38), II 2.08 (0.63, 0.75, 0.40, 0.30), III 1.98 (0.60, 0.68, 0.40, 0.30), IV 2.45 (0.75, 0.90, 0.50, 0.30). Carapace red-brown, with 2 clusters of white setae behind PLEs, 2 others posterolaterally on thoracic part, covered with tubercles and brown, thin setae. Fovea and radial groove indistinct. Chelicerae yellow, with 3 teeth on both the retromargin and promargin. Endites yellow. Labium darker than endites. Sternum sub-oval. Legs I robust, with inflated tibiae bearing dense ventral scales and 3 pairs of ventral spines; legs III and IV pale, with dark brown stripes prolaterally on femora, patellae and tibiae. Abdomen sub-oval, dorsum brown anteromedially, red-brown medio-posteriorly, with an irregular, transverse, yellow band bearing sparse, white lateral setae, covered entirely by a large scutum; venter dark brown posterolaterally.

Palp (Fig. 6A–D): femur about 2.5 times longer than wide; tibia almost as long as wide, with a thin, bar-shaped retrolateral apophysis slightly curved medially, blunt at tip; cymbium almost 2 times longer than wide in ventral view; bulb swollen; embolus fully coiled 2 times; process of embolic disc strongly curved medially, slightly pointed apically in retrolateral view.

**Female** (Fig. 7A, B, E). Total length 2.76. Carapace 1.26 long, 0.90 wide. Abdomen 1.40 long, 0.99 wide. Clypeus 0.06 high. Eye sizes and inter-distances: AME 0.31, ALE 0.12, PLE 0.11, AERW 0.75, PERW 0.79, EFL 0.57. Legs: I 2.04 (0.63, 0.78, 0.38, 0.25), II 1.63 (0.50, 0.60, 0.28, 0.25), III 1.56 (0.53, 0.53, 0.25, 0.25), IV 2.03 (0.65, 0.75, 0.38, 0.25). Habitus similar to that of male except darker and with less-developed legs I.

Epigyne (Fig. 7A, B): longer than wide, with a pair of lateral plates (described as stiffener in Azarkina and Haddad 2020) near the copulatory ducts; atrium elongate-oval, about 2 times longer than wide and separated by a narrow septum; copulatory openings almost C-shaped, medially located; copulatory ducts expanded proximally, then curved posteriorly into convoluted coils; spermathecae small, elongate-oval; fertilization ducts anterior to spermathecae, extending anterolaterally.

##### Distribution.

Known only from the type locality in Yunnan, China.

##### Comments.

Although not collected together, the male and female are considered to be conspecific because they share a very similar habitus.

#### Genus Myrmarachne

Taxon classificationAnimaliaAraneaeSalticidae

MacLeay, 1839

11E4F30C-3A8D-5140-B2D5-7F21BDE44CC5

##### Type species.

*Myrmarachnemelanocephala* MacLeay, 1839 from India.

#### Myrmarachne liui sp. nov.

Taxon classificationAnimaliaAraneaeSalticidae

E1F4A94F-0121-51DB-973A-BBE15873D9C6

http://zoobank.org/7E4DE420-AC1F-4D5E-9E9E-9DD8B6258477

Figs 8 , 9

##### Type material.

***Holotype*** ♂ (IZCAS-Ar42599), China: Yunnan: Xishuangbanna, Mengla County, Menglun Town, XTBG, tropical rainforest (21°55.20'N, 101°16.21'E, ca. 550 m), 07.viii.2018, C. Wang leg. ***Paratypes*** 1♀ (IZCAS-Ar42600), same data as holotype; 4♀1♂ (IZCAS-Ar42601–42605), site #3 of garbage dump off G213 roadside (21°54.34'N, 101°16.79'E, ca. 620 m), 2.v.2019, Y. Tong leg.; 5♀2♂ (IZCAS-Ar42606–42612), site #3 of garbage dump off G213 roadside (21°53.92'N, 101°16.01'E, ca. 560 m), 3.v.2019, Y. Tong leg.; 2♂ (IZCAS-Ar42613–42614), site #6 of garbage dump off G213 roadside (21°54.33'N, 101°16.79'E, ca. 620 m), 7.v.2019, Y. Tong leg.

##### Etymology.

The specific name is a patronym in honor of Mr. Hong Liu (Tongren, China), one of the collectors of the new species; noun (name) in genitive case.

##### Diagnosis.

**Figure 8.**
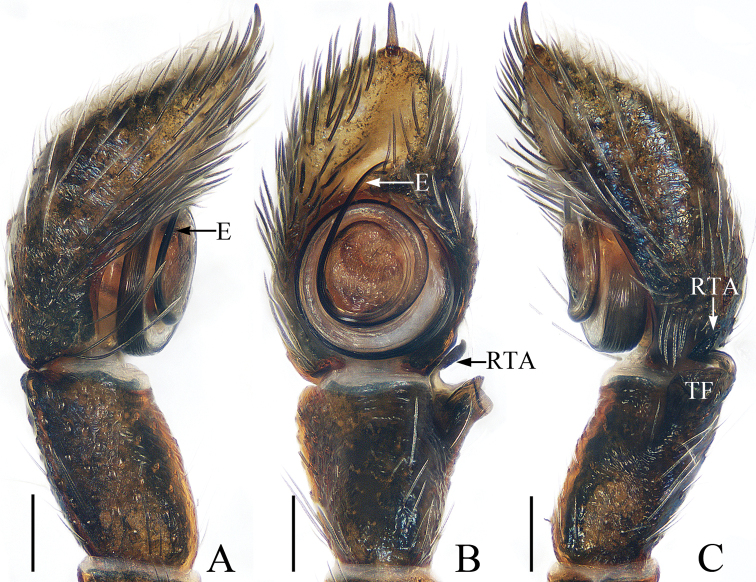
Male palp of *Myrmarachneliui* sp. nov., holotype **A** prolateral **B** ventral **C** retrolateral. Scale bars: 0.1.

**Figure 9.**
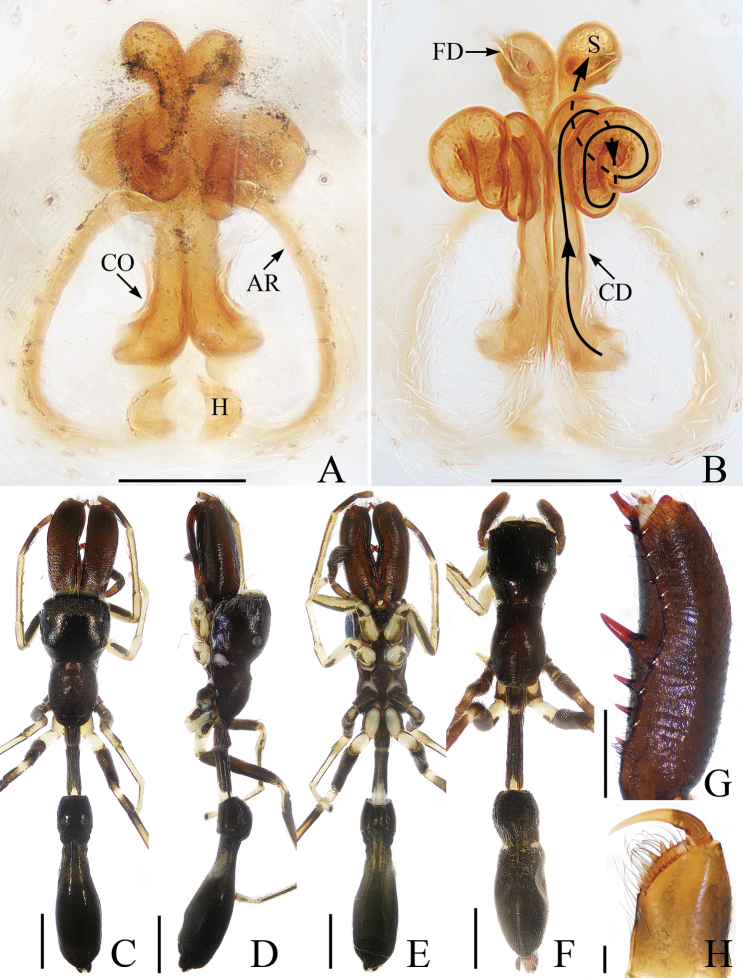
Female paratype and male holotype of *Myrmarachneliui* sp. nov. **A** epigyne, ventral **B** vulva, dorsal **C** holotype habitus, dorsal **D** lateral **E** ventral **F** female paratype habitus, dorsal **G** holotype left chelicera, posterior **H** female paratype left chelicera, posterior. Scale bars: 0.1 (**A, B, H**); 1.0 (**C–F**); 0.5 (**G**).

The male of *Myrmarachneliui* sp. nov. closely resembles that of *M.cornuta* Badcock, 1918 in having a similar habitus and palpal structure, but it differs by the following: 1) the second embolic coil is almost 2/3 the bulb width (Fig. 8B) vs. almost 4/5 the bulb width in *M.cornuta* (Edmunds and Prószyński 2003: fig. 33); 2) the retrolateral tibial apophysis is directed anteriorly in retrolateral view (Fig. 8B) vs. directed towards the bulb in *M.cornuta* (Edmunds and Prószyński 2003: fig. 35). The male is also similar to *M.contracta* (Karsch, 1880) in habitus and palpal structure, but it can be easily distinguished from the latter by having a large, truncated first apical promarginal cheliceral tooth (Fig. 9C, E, G) vs. absent in *M.contracta* (Edwards and Benjamin 2009: fig. 1D, H). The female of this new species is similar to *M.jianfenglin* Barrion, Barrion-Dupo & Heong, 2013 but differs by: 1) the epigynal hoods separate from each other by half their minimum width (Fig. 9A) vs. fused in *M.jianfenglin* (Barrion et al. 2013: fig. 25D); 2) the chelicerae, with seven promarginal and nine retromarginal teeth (Fig. 9H) vs. four promarginal and eight retromarginal teeth in *M.jianfenglin* (Barrion et al. 2013: fig. 25B).

##### Description.

**Male** (Figs 8, 9C–E, G). Total length 6.84. Carapace 2.41 long, 1.29 wide. Abdomen 3.15 long, 0.85 wide. Clypeus 0.07 high. Eye sizes and inter-distances: AME 0.41, ALE 0.24, PLE 0.22, AERW 1.22, PERW 1.24, EFL 0.92. Legs: I 3.77 (1.18, 1.63, 0.63, 0.33), II 2.98 (0.95, 1.15, 0.55, 0.33), III 3.29 (1.01, 1.15, 0.78, 0.35), IV 4.73 (1.45, 1.80, 1.13, 0.35). Carapace dark brown, strongly constricted between cephalic and thoracic parts, with clusters of white setae at lateral margins of constriction. Chelicerae well-developed, with 7 promarginal and 8 retromarginal teeth; first apical promarginal tooth truncated, the third one longest. Pedicel almost 2/5 abdomen length. Endites yellow-brown to dark brown. Labium colored as endites. Sternum dark, narrow, more than 3 times longer than wide. Legs I, II off-yellow, except metatarsi and tarsi red-brown, with dark brown femoral stripes laterally; legs III and IV off-yellow to dark brown. Abdomen elongated, constricted anteromedially; dorsum dark brown, with indistinct markings medially; venter slightly paler than dorsum.

Palp (Fig. 8A–C): femur about 3 times longer than wide; tibia longer than wide, with a sub-square flange and a sclerotized retrolateral apophysis curved and twisted into a pointed tip in retrolateral view; cymbium almost 1.5 times longer than wide in ventral view, and with an apical spine; bulb almost round, with tapered, S-shaped sperm duct; embolus coiled, tapered, with 2 circles.

**Female** (Fig. 9A, B, F, H). Total length 7.22. Carapace 2.70 long, 1.15 wide. Abdomen 2.78 long, 0.93 wide. Clypeus 0.07 high. Eye sizes and inter-distances: AME 0.40, ALE 0.20, PLE 0.20, AERW 1.12, PERW 1.20, EFL 0.88. Legs: I 3.19 (1.03, 1.33, 0.50, 0.33), II 2.76 (0.90, 1.08, 0.45, 0.33), III 3.24 (1.01, 1.13, 0.75, 0.35), IV 4.90 (1.50, 1.88, 1.12, 0.40). Habitus similar to that of male except chelicerae less developed, with 9 retromarginal teeth.

Epigyne (Fig. 9A, B): longer than wide, with pair of bell-shaped posterior hoods separated from each other by about 1/2 their minimum width; atrium almost trapeziform, with pair of arc-shaped lateral ridges; copulatory openings slit-shaped, located anteromedially on atrium; copulatory ducts membranous at origin, leading to a sclerotized part, slightly curved at base, then extending anteriorly along longitudinal axis, twisted into 2 loops, connected to oval spermathecae; fertilization ducts originate from posterior portion of spermathecae, lamellar.

##### Distribution.

Known only from the type locality in Yunnan, China.

#### Genus Nandicius

Taxon classificationAnimaliaAraneaeSalticidae

Prószyński, 2016

280F8F09-244F-5EEB-9A6A-2022C4E56C0C

##### Type species.

*Phintellamussooriensis* Prószynski, 1992 from India.

#### Nandicius proszynskii sp. nov.

Taxon classificationAnimaliaAraneaeSalticidae

80F36DCA-A969-590E-B809-366B9CDA3385

http://zoobank.org/B828C473-DAD5-4F74-A04C-18594EFBA2C7

Figs 10 , 11

##### Type material.

***Holotype*** ♂ (IZCAS-Ar42615), China: Yunnan: Xishuangbanna, Mengla County, Menglun Town, XTBG, Grove (21°53.84'N, 101°16.48'E, ca. 550 m), 10.v.2019, H. Yu leg. ***Paratype*** 1♂ (IZCAS-Ar42616), same data as holotype.

##### Etymology.

The species name is a patronym in honor of Prof. Jerzy Prószyński (Warsaw, Poland), who has made significant contributions to the taxonomy of salticid spiders; noun (name) in genitive case.

##### Diagnosis.

*Nandiciusproszynskii* sp. nov. can be easily distinguished from any congeners by the presence of a disto-retrolateral femoral apophysis and a short retrolateral tibial apophysis, which is shorter than the dorsal tibial apophysis vs. lacking a femoral apophysis and with a retrolateral tibial apophysis longer than the dorsal tibial apophysis.

##### Description.

**Figure 10.**
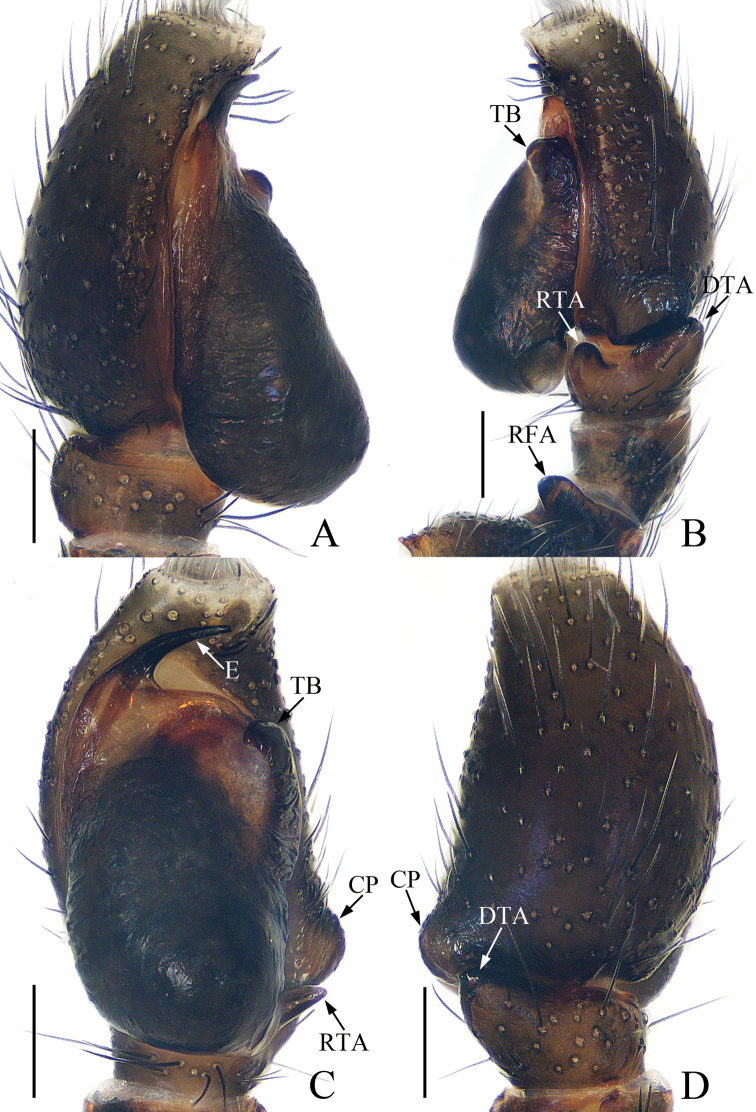
Male palp of *Nandiciusproszynskii* sp. nov., holotype **A** prolateral **B** retrolateral **C** ventral **D** dorsal. Scale bars: 0.1.

**Figure 11.**
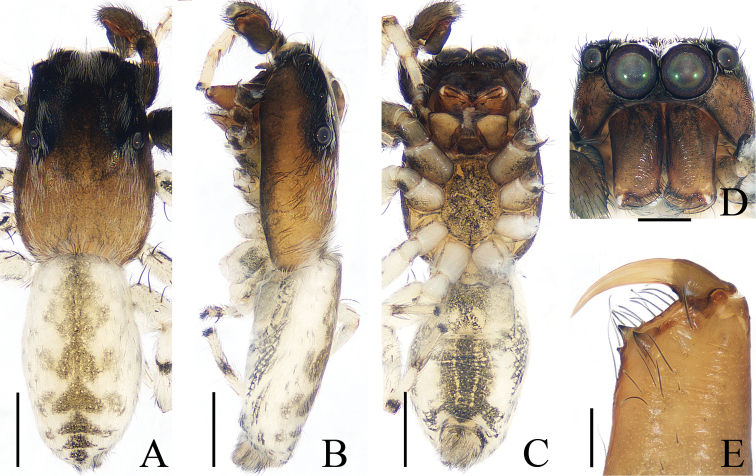
*Nandiciusproszynskii* sp. nov., holotype **A** habitus, dorsal **B** lateral **C** ventral **D** carapace, frontal **E** left chelicera, posterior. Scale bars: 0.5 (**A–C**); 0.2 (**D**); 0.1 (**E**).

**Male** (Figs 10, 11). Total length 2.75. Carapace 1.46 long, 0.98 wide. Abdomen 1.44 long, 0.83 wide. Clypeus 0.01 high. Eye sizes and inter-distances: AME 0.24, ALE 0.14, PLE 0.13, AERW 0.76, PERW 0.82, EFL 0.63. Legs: I 2.09 (0.63, 0.85, 0.37, 0.24); II 1.61 (0.51, 0.59, 0.27, 0.24); III 1.71 (0.54, 0.59, 0.34, 0.24); IV 2.24 (0.76, 0.83, 0.41, 0.24). Carapace yellow to dark brown, slightly narrowed anteromedially, covered by dense, white and brown setae. Fovea indistinct. Chelicerae yellow, with 1 retromarginal tooth and 2 promarginal teeth. Endites yellow to pale brown. Labium dark brown, linguiform. Sternum yellow, mingled with green-brown, with white setae. Legs pale yellow to yellow except femora I dark brown. Abdomen elongated, dorsum pale yellow laterally, with an irregular, longitudinal, green-brown stripe over entire surface; venter pale yellow laterally, brown medially, with pair of dotted lines.

Palp (Fig. 10A–D): femur about 2.5 times longer than wide, with digitiform disto-retrolateral apophysis; tibia wider than long, with short, blunt retrolateral apophysis, and a lobe-shaped dorsal apophysis; cymbium about 1.8 times longer than wide in ventral view, with a swollen baso-retrolateral process; bulb inflated, with a flat, disto-retrolateral tegular bump; embolus sclerotized, originating at ~11 o’clock position of bulb, tapered, slightly curved to a pointed tip, directed retrolaterally.

**Female.** Unknown.

##### Distribution.

Known only from the type locality in Yunnan, China.

#### Genus Phintelloides

Taxon classificationAnimaliaAraneaeSalticidae

Kanesharatnam & Benjamin, 2019

E2BD7934-AA17-5F84-81B2-609D3590A415

##### Type species.

*Chrysillajesudasi* Caleb & Mathai, 2014 from India.

#### Phintelloides pengi sp. nov.

Taxon classificationAnimaliaAraneaeSalticidae

89FAA543-5A5A-53CF-BD22-8B8826706ADB

http://zoobank.org/DC8905E5-825B-4502-9695-C19E5027BAEF

Figs 12 , 13

##### Type material.

***Holotype*** ♂ (IZCAS-Ar42617), China: Yunnan: Xishuangbanna, Mengla County, Menglun Town, site #5 of garbage dump off G213 roadside (21°54.37'N, 101°16.70'E, ca. 620 m), 6.v.2019. Y. Tong leg. ***Paratypes*** 1♀ (IZCAS-Ar42618), garbage dump off G213 roadside (21°54.38'N, 101°16.82'E, 630 m), 23.xi.2009, G. Tang, Z. Yao leg.; 3♂ (IZCAS-Ar42619–42621), Masuoxing Village (21°54.02'N, 101°16.90'E, ca. 560 m), 27.04.2019, Y. Tong leg.; 1♀ (IZCAS-Ar42622), secondary tropical seasonal moist forest (21°54.72'N, 101°16.94'E, ca. 660 m), 1–15.iii.2007, G. Zheng leg.

##### Etymology.

The species name is a patronym in honor of Prof. Xianjin Peng (Changsha, China), who has made significant contributions to the taxonomy of Chinese salticids; noun (name) in genitive case.

##### Diagnosis.

**Figure 12.**
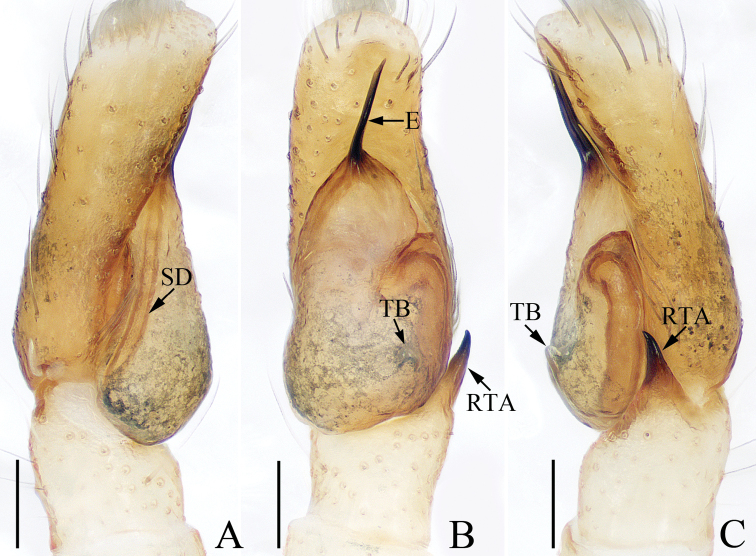
Male palp of *Phintelloidespengi* sp. nov., holotype **A** prolateral **B** ventral **C** retrolateral. Scale bars: 0.1.

**Figure 13.**
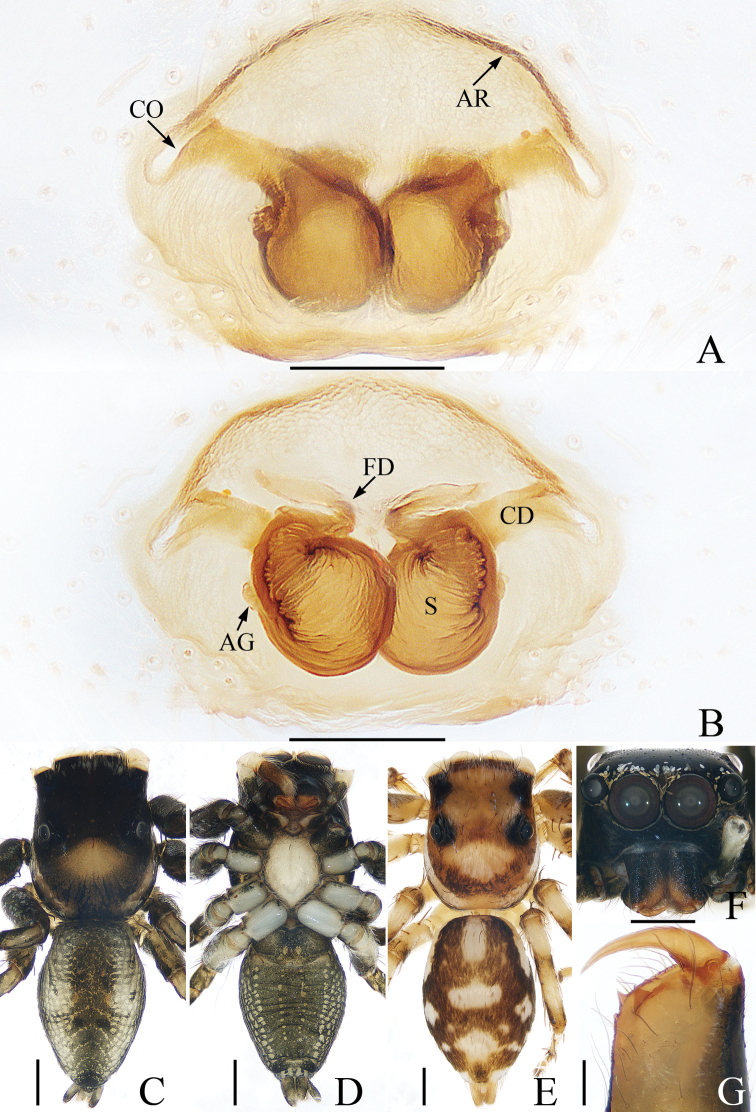
Female paratype and male holotype of *Phintelloidespengi* sp. nov. **A** epigyne, ventral **B** vulva, dorsal **C** holotype habitus, dorsal **D** ventral **E** female paratype habitus, dorsal **F** holotype carapace, frontal **G** holotype left chelicera, posterior. Scale bars: 0.1 (**A, B, G**); 0.5 (**C–F**).

The new species resembles *Phintelloidesversicolor* (C. L. Koch, 1846) and *Phintellaleucaspis* (Simon, 1903) in the copulatory organs, but it can be easily distinguished by the straight embolus (Fig. 12B) vs. curved in *P.versicolor* and *Phintellaleucaspis* (Żabka, 1985: figs 83, 88, 91 and Bohdanowicz and Prószyński 1987: fig. 214) and the laterally located, widely separated copulatory openings, separated from each other by more than 3 times the spermathecal width (Fig. 13A) vs. anteriorly located copulatory openings, separated slightly more than 2 times the spermathecal width in *P.versicolor* (Żabka, 1985: fig. 93).

##### Description.

Male (Figs 12, 13C, D, F, G). Total length 3.57. Carapace 1.71 long, 1.40 wide. Abdomen 1.83 long, 1.19 wide. Clypeus 0.10 high. Eye sizes and inter-distances: AME 0.42, ALE 0.23, PLE 0.22, AERW 1.25, PERW 1.21, EFL 0.89. Legs: I 3.73 (1.15, 1.45, 0.75, 0.38), II 3.49 (1.13, 1.23, 0.75, 0.38), III 3.94 (1.25, 1.28, 0.98, 0.43), IV 4.22 (1.33, 1.45, 1.01, 0.43). Carapace dark brown, with a fan-shaped yellow area on anterior thoracic part and pair of yellow lateral bands with white scales, covered with white and yellow scales anteriorly. Fovea longitudinal, red, thin, bar shaped. Chelicerae yellow-red to dark brown, with 1 retromarginal tooth and 2 promarginal teeth. Endites dark, somewhat greenish, pale entally. Labium colored as endites. Sternum pale, with white and brown setae. Legs green-brown to dark brown except tarsi, metatarsi yellow and coxae, trochanter of legs II, III, IV pale. Abdomen sub-oval, dorsum gray-white to dark green, pale and dotted laterally, with longitudinal dark green band over entire surface medially; venter colored as dorsum, with pair of dotted lines medially.

Palp (Fig. 12A–C): femur about 2.5 times longer than wide, dark brown proximal half; tibia almost as long as wide, with tapered retrolateral tibial apophysis, broad basally, slightly curved distally, pointed apically; cymbium about 2.5 times longer than wide in ventral view, bearing white scales dorsally; bulb inflated, with digitiform baso-retrolateral tegular bump; embolus straight, longer than retrolateral tibial apophysis, originating from apical portion of bulb, tip directed towards about 12:30 o’clock in ventral view.

**Female** (Fig. 13A, B, E) Total length 4.27. Carapace 1.77 long, 1.56 wide. Abdomen 2.26 long, 1.51 wide. Clypeus 0.11 high. Eye sizes and inter-distances: AME 0.44, ALE 0.26, PLE 0.25, AERW 1.42, PERW 1.39, EFL 1.03. Legs: I 3.74 (1.18, 1.43, 0.73, 0.40), II 3.70 (1.15, 1.42, 0.73, 0.40), III 4.19 (1.33, 1.40, 1.01, 0.45), IV 4.71 (1.50, 1.63, 1.13, 0.45). Carapace similar to that of male except pale. Abdomen dark brown dorsally, with 3 longitudinal, pale bands anteriorly, 3 transverse, pale bands medially, irregular pale markings and dots posteriorly, pair of pale spots at terminus.

Epigyne (Fig. 13A, B) slightly wider than long; atrium large, with an arc-shaped anterior ridge; copulatory openings slit-shaped, located at lateral edges of atrial ridge, separated by almost 3 times the spermathecal width; copulatory ducts short, straight, oblique, connected to anterior edges of spermathecae, with short accessory glands at terminus; spermathecae almost oval, overlapping entally, anteriorly with tube-shaped extensions connected to lamellar fertilization ducts.

##### Distribution.

Known only from the type locality in Yunnan, China.

##### Comments.

The male and female are considered to be the same species because they were collected from the same site without other candidates and have copulatory organs similar to *Phintelloidesversicolor*. *Phintellaleucaspis* (Simon, 1903) shares a very similar palp with *Phintelloidesversicolor* and the new species, thus it most likely belongs to *Phintelloides*.

#### Genus Poecilorchestes

Taxon classificationAnimaliaAraneaeSalticidae

Simon, 1901

B3943104-9F90-551D-85C4-A2BDBB9E743B

##### Type species.

*Poecilorchestesdecoratus* Simon, 1901 from New Guinea.

#### Poecilorchestes zhengi sp. nov.

Taxon classificationAnimaliaAraneaeSalticidae

47B7ADC5-DBFE-528A-B4A5-0C4478A333A0

http://zoobank.org/3E6F0834-1F51-491E-A8E9-46EFC38007CB

Figs 14 , 15

##### Type material.

***Holotype*** ♂ (IZCAS-Ar42623), China: Yunnan: Xishuangbanna, Mengla County, Menglun Town, secondary tropical seasonal moist forest (21°54.72'N, 101°16.94'E, ca. 660 m), 27.vii.2007, G. Zheng leg. ***Paratypes*** 2♀1♂ (IZCAS-Ar42624–42626), same data as holotype.

##### Etymology.

The species name is a patronym in honor of Prof. Guo Zheng (Shenyang, China), the collector of the new species; noun (name) in genitive case.

##### Diagnosis.

**Figure 14.**
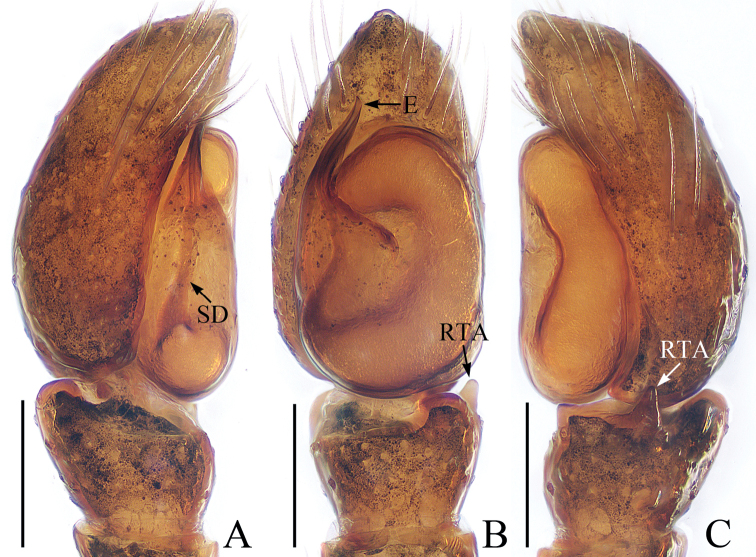
Male palp of *Poecilorchesteszhengi* sp. nov., holotype **A** prolateral **B** ventral **C** retrolateral. Scale bars: 0.1.

**Figure 15.**
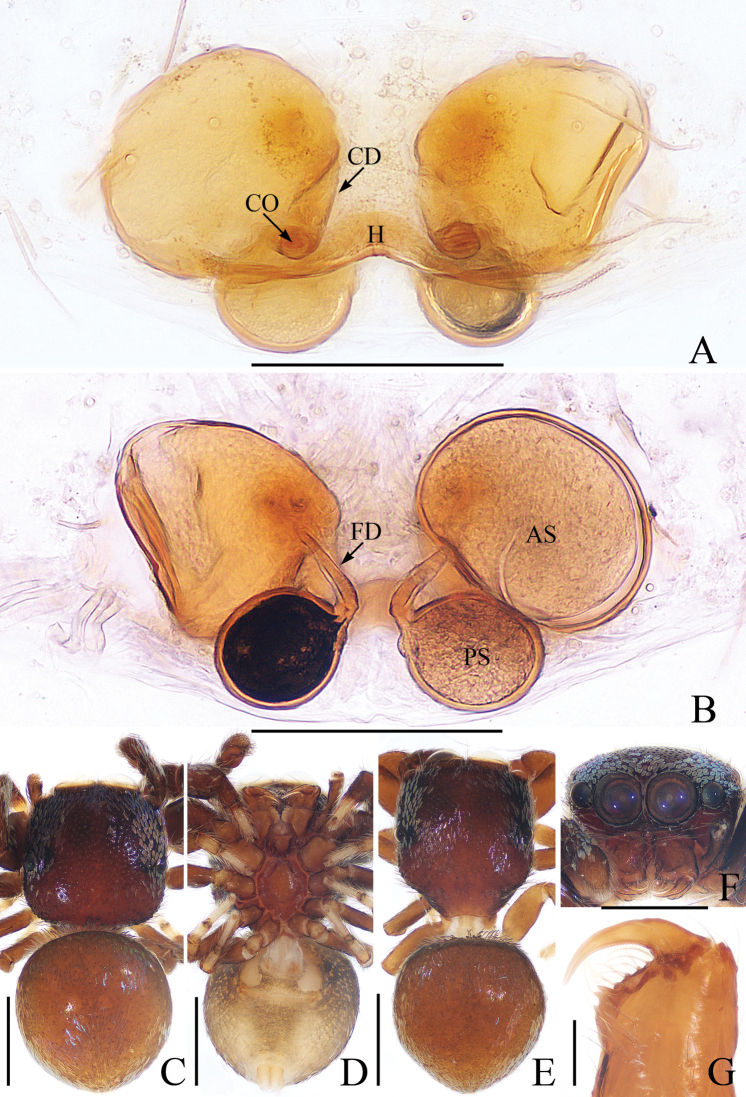
Female paratype and male holotype of *Poecilorchesteszhengi* sp. nov. **A** epigyne, ventral **B** vulva, dorsal **C** holotype habitus, dorsal **D** ventral **E** female paratype habitus, dorsal **F** holotype carapace, frontal **G** holotype left chelicera, posterior. Scale bars: 0.1 (**A, B, G**); 0.5 (**C–F**).

The male of *Poecilorchesteszhengi* sp. nov. resembles that of *P.logunovi* Prószyński & Deeleman-Reinhold, 2013 in having a square carapace bearing scales and a similar palp, but it can be easily distinguished by the visible retrolateral tibial apophysis and the thicker sperm duct with a maximum diameter almost half of bulb width in ventral view (Fig. 14B) vs. not visible and thinner sperm duct with a maximum diameter almost one-third of bulb width in *P.logunovi* (Prószyński and Deeleman-Reinhold 2013: fig. 115). The female resembles that of *Stertiniusryukyuensis* Suguro, 2020 in the general shape of the epigyne but differs in the following: 1) the epigyne has a central hood (Fig. 15A) vs. a central fold in *S.ryukyuensis* (Suguro 2020: fig. 15); 2) the copulatory openings are almost slit-shaped (Fig. 15A) vs. oval in *S.ryukyuensis* (Suguro 2020: fig. 15); 3) the presence of a dorsal abdominal scutum (Fig. 15E) vs. absent in *S.ryukyuensis* (Suguro 2020: fig. 2).

##### Description.

**Male** (Figs 14, 15C, D, F, G). Total length 1.69. Carapace 0.78 long, 0.81 wide. Abdomen 0.90 long, 0.88 wide. Clypeus 0.03 high. Eye sizes and inter-distances: AME 0.25, ALE 0.12, PLE 0.11, AERW 0.72, PERW 0.81, EFL 0.46. Legs: I 1.61 (0.58, 0.65, 0.20, 0.18), II 1.21 (0.40, 0.45, 0.18, 0.18), III 1.01 (0.38, 0.30, 0.15, 0.18), IV 1.18 (0.45, 0.40, 0.15, 0.18). Carapace square, red-brown, covered with multicolor scales, denser anteriorly and laterally. Fovea indistinct. Chelicerae yellow-red, with 2 promarginal teeth and 1 retromarginal tooth bifurcated into pointed tips. Endites colored as chelicerae. Labium and sternum red-brown. Legs I red-brown, except tarsi yellow, with inflated femora, and tibial scales; other legs red-brown with pale yellow metatarsi and tarsi. Abdomen almost round, dorsum paler than carapace, bearing scales and brown, thin setae, covered entirely by a scutum; venter pale to brown, covered with thin, brown setae.

Palp (Fig. 14A–C): femur about 2.5 times longer than wide; tibia slightly wider than long; retrolateral tibial apophysis less than 1/3 tibial length, with broad ventral extension and blunt tip; cymbium about 1.8 times longer than wide in ventral view; bulb sub-oval; sperm duct broad, with maximum diameter almost 1/2 of bulb width; embolus originating from ~9:30 o’clock position of bulb, slightly curved, with blunt tip.

**Female** (Fig. 15A, B, E). Total length 1.79. Carapace 0.82 long, 0.75 wide. Abdomen 0.85 long, 0.85 wide. Clypeus 0.03 high. Eye sizes and inter-distances: AME 0.24, ALE 0.11, PLE 0.10, AERW 0.69, PERW 0.76, EFL 0.43. Legs: I 1.23 (0.40, 0.50, 0.15, 0.18), II 1.04 (0.35, 0.38, 0.13, 0.18), III 0.96 (0.33, 0.30, 0.15, 0.18), IV 1.11 (0.43, 0.35, 0.15, 0.18). Habitus similar to that of male.

Epigyne (Fig. 15A, B): wider than long, with bell-shaped hood medially; copulatory openings slit-shaped, lateral to epigynal hood; copulatory ducts short, about equal length to width of posterior chamber of spermathecae, connected medially to ental sides of anterior chamber of spermathecae; spermathecae divided into two sub-spherical chambers; fertilization ducts originate from apico-ental portion of posterior chamber of spermathecae.

##### Distribution.

Known only from the type locality in Yunnan, China.

##### Comments.

The species is temporarily placed into *Poecilorchestes* due to its general resemblance to *P.logunovi* Prószyński & Deeleman-Reinhold, 2013. Further data are required to confirm the placement of this species.

#### Genus Rhene

Taxon classificationAnimaliaAraneaeSalticidae

Thorell, 1869

A34E005E-2FF9-50B4-AD2A-D7C85974BEB7

##### Type species.

*Rhanisflavigera* C. L. Koch, 1846 from Indonesia.

#### Rhene wandae sp. nov.

Taxon classificationAnimaliaAraneaeSalticidae

34AD9FC6-3E44-5F9E-B89D-A0FB81B6E9DB

http://zoobank.org/313F1981-ECB3-484D-A5A6-EFD9D803ADB4

Figs 16 , 17

##### Type material.

***Holotype*** ♂ (IZCAS-Ar42627), China: Yunnan: Xishuangbanna, Mengla County, Menglun Town, Lvshilin (21°54.71'N, 101°16.90'E, ca. 660 m) 13.xi.2009, G. Tang, Z. Yao leg. ***Paratype*** 1♂ (IZCAS-Ar42628), Bamboo plantation off G213 roadside (21°53.64'N, 101°16.94'E, ca. 580 m), 13.xi.2009, G. Tang, Z. Yao leg.

##### Etymology.

The species name is a patronym in honor of Prof. Wanda Wesołowska (Wrocław, Poland), who has contributed significantly to the taxonomy of the genus *Rhene*; noun (name) in genitive case.

##### Diagnosis.

The new species can be easily distinguished from any congeners by the coiled embolus which is longer than the bulb vs. embolus markedly shorter than the bulb, straight or curved in other species.

##### Description.

**Figure 16.**
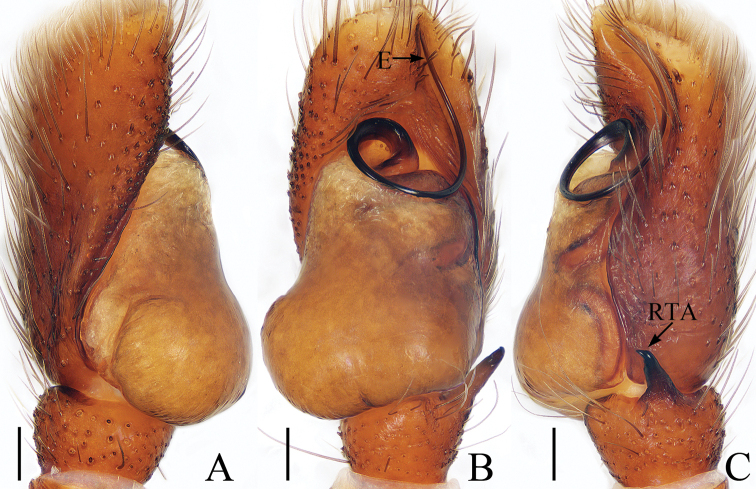
Male palp of *Rhenewandae* sp. nov., holotype **A** prolateral **B** ventral **C** retrolateral. Scale bars: 0.1.

**Figure 17.**
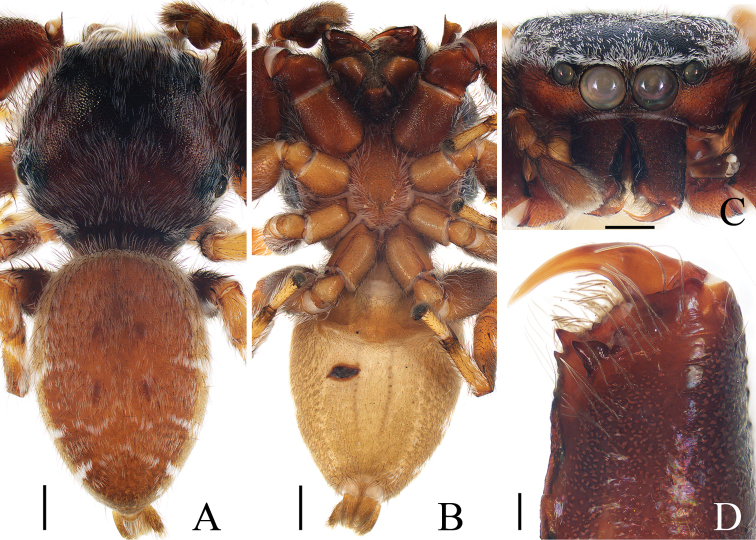
*Rhenewandae* sp. nov., holotype **A** habitus, dorsal **B** ventral **C** carapace, frontal **D** chelicera, posterior. Scale bars: 0.5 (**A–C**); 0.1 (**D**).

**Male** (Figs 16, 17). Total length 5.26. Carapace 2.40 long, 2.41 wide. Abdomen 2.98 long, 2.05 wide. Clypeus 0.02 high. Eye sizes and inter-distances: AME 0.47, ALE 0.26, PLE 0.24, AERW 1.53, PERW 2.38, EFL 1.58. Legs: I 6.02 (2.01, 2.43, 0.98, 0.60), II 4.11 (1.38, 1.55, 0.73, 0.45), III 3.68 (1.23, 1.25, 0.75, 0.45), IV 4.85 (1.50, 1.95, 0.95, 0.45). Carapace almost elongated-hexagonal, dark red to dark brown, covered with dense, brown and off-white setae. Fovea dark brown, longitudinal. Chelicerae red-brown, with 1 retrolateral and 2 promarginal teeth. Endites colored as chelicerae, with brown setae entally on the medio-distal margin. Labium slightly darker than endites. Sternum yellow-brown, elongate-oval, covered by off-white, thin setae. Legs I red-brown, with inflated tibiae ventrally with dense, brown setae, other legs yellow-brown. Abdomen sub-oval, dorsum with 2 pairs of muscle depressions medially and irregular, transverse, white stripes of setae medio-posteriorly, covered with dark and off-white thin setae; venter yellow-gray, with a pair of medial dotted lines.

Palp (Fig. 16A–C): femur about 2 times longer than wide; tibia wider than long, with a sclerotized, tapered, retrolateral apophysis slightly shorter than tibial length, distally curved to a pointed tip directed ventrally in retrolateral view; cymbium about 1.8 times longer than wide in ventral view, covered with dense setae; bulb swollen; embolus long, tapered, coiled nearly 1.5×, with a rather pointed tip, reaches cymbial tip.

**Female.** Unknown.

##### Distribution.

Known only from the type locality in Yunnan, China.

#### Genus Simaetha

Taxon classificationAnimaliaAraneaeSalticidae

Thorell, 1881

58894296-7E92-5A7B-A10B-D62CA7707F82

##### Type species.

*Simaethathoracica* Thorell, 1881 from Australia.

#### Simaetha cheni sp. nov.

Taxon classificationAnimaliaAraneaeSalticidae

4FAC6EB7-47F4-5949-A88F-99B90A53E6BB

http://zoobank.org/C305862D-D9A4-447D-A7D7-5B34A7024926

Figs 18 , 19

##### Type material.

***Holotype*** ♂ (IZCAS-Ar42629), China: Yunnan: Xishuangbanna, Mengla County, Menglun Town, site #1 of garbage dump off G213 roadside (21°54.28'N, 101°16.75'E, ca. 630 m), 25.iv.2019, Z. Chen leg. ***Paratypes*** 1♀ (IZCAS-Ar42630), same data as holotype; 1♀ (IZCAS-Ar42631), site #4 of garbage dump off G213 roadside (21°54.34'N, 101°16.79'E, ca. 618 m), 3.v.2019, Y. Tong leg.; 1♀1♂ (IZCAS-Ar42632–42633), site #6 of garbage dump off G213 roadside (21°54.33'N, 101°16.79'E, ca. 620 m), 7.v.2019, Y. Tong leg.

##### Etymology.

The species name is a patronym in honor of Mr. Zhigang Chen (Beijing, China), one of the collectors of the new species; noun (name) in genitive case.

##### Diagnosis.

**Figure 18.**
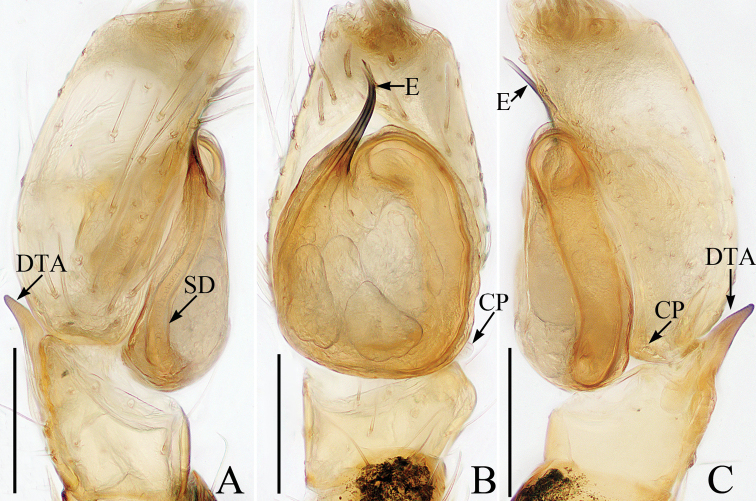
Male palp of *Simaethacheni* sp. nov., holotype **A** prolateral **B** ventral **C** retrolateral. Scale bars: 0.1.

**Figure 19.**
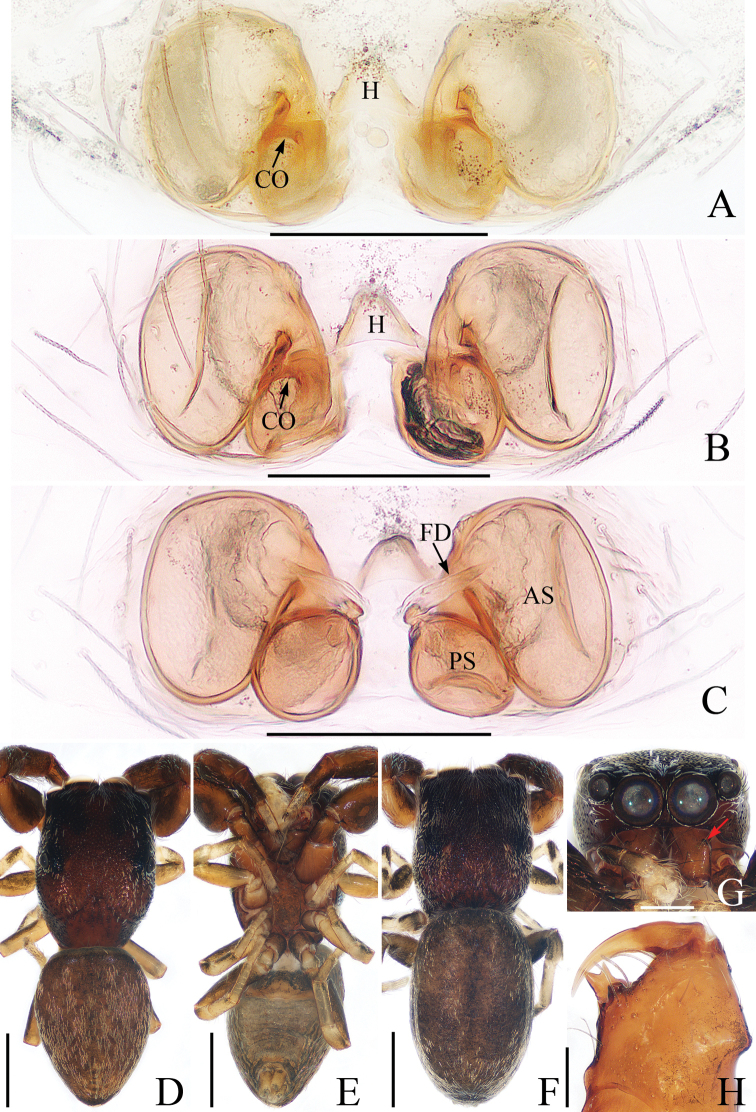
Female paratype and male holotype of *Simaethacheni* sp. nov. **A, B** epigyne, ventral **C** vulva, dorsal **D** holotype habitus, dorsal **E** ventral **F** female paratype habitus, dorsal **G** holotype carapace, frontal **H** holotype right chelicera, anterior. Scale bars: 0.1 (**A–C, H**); 0.5 (**D–F**); 0.2 (**G**).

The male of *Simaethacheni* sp. nov. resembles that of *S.menglun* Wang & Li, 2020 by the similar palp, but it can be easily distinguished by the following: 1) the embolus is slightly curved into a blunt tip directed anteriorly in ventral view (Fig. 18B) vs. strongly curved into a pointed tip directed prolaterally in *S.menglun* (Wang and Li 2020: fig. 11C); 2) the chelicerae have a process mediolaterally on the anterior surface of the paturon (Fig. 19G, H) vs. process absent in *S.menglun* (Wang and Li 2020: fig. 12G). The female of this species resembles that of *S.broomei* Żabka, 1994 in having a similar epigyne, but it can be easily distinguished by the epigynal hood, which is less than 2/3 the width of the posterior chamber of the spermathecae (Fig. 19A–C) vs. about 8/6 the width of the posterior chamber of the spermathecae (Żabka 1994: fig. 18B, C).

##### Description.

**Male** (Figs 18, 19D, E, G, H). Total length 2.18. Carapace 1.11 long, 0.82 wide. Abdomen 1.10 long, 0.79 wide. Clypeus 0.01 high. Eye sizes and inter-distances: AME 0.24, ALE 0.12, PLE 0.11, AERW 0.71, PERW 0.80, EFL 0.56. Legs: I 1.66 (0.60, 0.63, 0.23, 0.20), II 1.35 (0.45, 0.45, 0.25, 0.20), III 1.25 (0.40, 0.40, 0.25, 0.20), IV 1.63 (0.58, 0.55, 0.30, 0.20). Carapace red-brown, with pair of round, dark spots mediolaterally in eye field, covered with short, brown setae and off-white scales. Chelicerae yellow-red, with a process mediolaterally on anterior surface of paturon, 2 promarginal teeth, 1 pillar-shaped tooth with 2 pointed tips. Endites red-brown. Labium darker than endites. Sternum elongate, 2 times longer than wide. Legs I red-brown to dark brown except tarsi yellow, with inflated femora, and fluorescent-green scales dorsally and laterally on patellae and tibiae; other legs yellow-brown, femora darker prolaterally. Abdomen sub-oval, dorsum dark brown, covered entirely by a large scutum with dense scales; venter brown, mingled with green.

Palp (Fig. 18A–C): femur about 2.5 times longer than wide; tibia wider than long, with a tapered dorsal apophysis slightly shorter than tibial length, directed towards about 1:30 o’clock apically in retrolateral view; cymbium about 1.5 times longer than wide in ventral view, with a sub-triangular baso-retrolateral process; bulb almost round; embolus originating at 10: 30 o’clock position of bulb, slightly curved distally, with a rather blunt tip.

**Female** (Fig. 19A–C, F). Total length 2.22. Carapace 0.98 long, 0.81 wide. Abdomen 1.32 long, 0.84 wide. Clypeus 0.01 high. Eye sizes and inter-distances: AME 0.24, ALE 0.12, PLE 0.11, AERW 0.72, PERW 0.79, EFL 0.54. Legs: I 1.69 (0.60, 0.66, 0.23, 0.20), II 1.35 (0.45, 0.45, 0.25, 0.20), III 1.33 (0.45, 0.43, 0.25, 0.20), IV 2.04 (0.68, 0.68, 0.45, 0.23). Habitus similar to that of male except with a longer abdomen.

Epigyne (Fig. 19A–C) with central triangular hood almost 1.5× wider than long; copulatory openings arc shaped; copulatory ducts short; spermathecae divided into two chambers, anterior chamber bigger, almost reniform, posterior chamber almost spherical, separated by less than the width of hood; fertilization ducts originate from the apical portion of the posterior chamber of spermathecae.

##### Distribution.

Known only from the type locality in Yunnan, China.

## Supplementary Material

XML Treatment for Chinattus

XML Treatment for Chinattus inflatus

XML Treatment for Euochin

XML Treatment for Euochin yaoi

XML Treatment for Laufeia

XML Treatment for Laufeia banna

XML Treatment for Marengo

XML Treatment for Marengo tangi

XML Treatment for Myrmarachne

XML Treatment for Myrmarachne liui

XML Treatment for Nandicius

XML Treatment for Nandicius proszynskii

XML Treatment for Phintelloides

XML Treatment for Phintelloides pengi

XML Treatment for Poecilorchestes

XML Treatment for Poecilorchestes zhengi

XML Treatment for Rhene

XML Treatment for Rhene wandae

XML Treatment for Simaetha

XML Treatment for Simaetha cheni
